# Impact of autofluorescence for detection of parathyroid glands during thyroidectomy on postoperative parathyroid hormone levels: parallel multicentre randomized clinical trial

**DOI:** 10.1093/bjs/znad278

**Published:** 2023-09-27

**Authors:** Anders Bergenfelz, Marcin Barczynski, Anette Heie, Andreas Muth, Christian Passler, Max Schneider, Paulina Wierzbicka, Alexander Konturek, Katrin Brauckhoff, Anna-Karin Elf, Jakob Dahlberg, Michael Hermann

**Affiliations:** Department of Clinical Sciences—Lund, Medical Faculty, Lund University, Lund, Sweden; Department of Endocrine Surgery, Jagiellonian University Medical College, Krakow, Poland; Department of Breast and Endocrine Surgery, Haukeland University Hospital, Bergen, Norway; Department of Surgery, Sahlgrenska University Hospital, Gothenburg, Sweden; Department of Surgery, Institute of Clinical Sciences, Sahlgrenska Academy, University of Gothenburg, Gothenburg, Sweden; Department of Surgery, Klinik Landstraße, Wiener Gesundheitsverbund, Vienna, Austria; Department of Surgery, Klinik Landstraße, Wiener Gesundheitsverbund, Vienna, Austria; Department of Endocrine Surgery, Jagiellonian University Medical College, Krakow, Poland; Department of Endocrine Surgery, Jagiellonian University Medical College, Krakow, Poland; Department of Breast and Endocrine Surgery, Haukeland University Hospital, Bergen, Norway; Department of Clinical Science, University of Bergen, Bergen, Norway; Department of Surgery, Sahlgrenska University Hospital, Gothenburg, Sweden; Department of Surgery, Institute of Clinical Sciences, Sahlgrenska Academy, University of Gothenburg, Gothenburg, Sweden; Department of Surgery, Sahlgrenska University Hospital, Gothenburg, Sweden; Department of Surgery, Institute of Clinical Sciences, Sahlgrenska Academy, University of Gothenburg, Gothenburg, Sweden; Department of Surgery, Klinik Landstraße, Wiener Gesundheitsverbund, Vienna, Austria

## Abstract

**Background:**

Techniques for autofluorescence have been introduced to visualize the parathyroid glands during surgery and to reduce hypoparathyroidism after thyroidectomy.

**Methods:**

This parallel multicentre RCT investigated the use of Fluobeam^®^ LX to visualize the parathyroid glands by autofluorescence during total thyroidectomy compared with no use. There was no restriction on the indication for surgery. Patients were randomized 1 : 1 and were blinded to the group allocation. The hypothesis was that autofluorescence enables identification and protection of the parathyroid glands during thyroidectomy. The primary endpoint was the rate of low parathyroid hormone (PTH) levels the day after surgery.

**Results:**

Some 535 patients were randomized, and 486 patients received an intervention according to the study protocol, 246 in the Fluobeam^®^ LX group and 240 in the control group. Some 64 patients (26.0 per cent) in the Fluobeam^®^ LX group and 77 (32.1 per cent) in the control group had low levels of PTH after thyroidectomy (*P* = 0.141; relative risk (RR) 0.81, 95 per cent c.i. 0.61 to 1.07). Subanalysis of 174 patients undergoing central lymph node clearance showed that 15 of 82 (18 per cent) in the Fluobeam^®^ LX group and 31 of 92 (33 per cent) in the control group had low levels of PTH on postoperative day 1 (*P* = 0.021; RR 0.54, 0.31 to 0.93). More parathyroid glands were identified during operation in patients who had surgery with Fluobeam^®^ LX, and fewer parathyroid glands in the surgical specimen on definitive histopathology. No specific harm related to the use of Fluobeam^®^ LX was reported.

**Conclusion:**

The use of autofluorescence during thyroidectomy did not reduce the rate of low PTH levels on postoperative day 1 in the whole group of patients. It did, however, reduce the rate in a subgroup of patients. Registration number: NCT04509011 (http://www.clinicaltrials.gov).

## Teaser

This multicentre RCT, performed in four centres and in four countries, has provided evidence for the benefit of using autofluorescence during thyroidectomy to identify and preserve the parathyroid glands.

## Introduction

Temporary and permanent hypoparathyroidism are arguably the most common and important complications after total thyroidectomy. National studies^1–4^ have indicated that the risk of permanent postoperative hypoparathyroidism is 5–16 per cent, depending on definition and diagnosis, or even higher^5^. A recent systematic review^6^, including 45 studies with 23 164 patients, estimated the incidence of temporary hypoparathyroidism to be 29 per cent, and that of permanent hypoparathyroidism to be 4.1 per cent measured at 6 months after surgery. Analysis of national data has shown that patients treated for permanent hypoparathyroidism have an increased risk of morbidity^7^ and death^8^.

The use of near-infrared (NIR) autofluorescence was introduced a decade ago, and proposed as a means of identification and protection of the parathyroid glands during thyroid surgery^9,10^. A further advance in the area was to combine autofluorescence of the parathyroid glands with fluorescence imaging using indocyanine green (ICG) angiography. It was demonstrated that at least one well vascularized parathyroid gland, as assessed by ICG angiography^11,12^, predicted the absence of postoperative hypoparathyroidism^13^. The introduction of this technique in one centre was suggested to lower the risk of postoperative hypoparathyroidism on the first day after thyroidectomy^14^. It is not known, however, whether this was due to a general modification of surgical technique, whereby a small remnant of thyroid tissue was left in some patients, or an increased awareness of complications owing to the introduction of ICG angiography (Hawthorne effect)^12^.

A few RCTs have been performed with the use of NIR autofluorescence^15–18^ or NIR autofluorescence and ICG angiography^19,20^. Some of these RCTs were, however, either single-centre studies^16–20^, had small patient numbers^16,18,20^, or lacked a power calculation for the primary outcome^17,19^. The RCT reported by Benmiloud *et al.*^15^ was a multicentre study from three French centres, with a power calculation based on reduction of postoperative hypocalcaemia (defined as calcium level below 2.00 mmol/l).

The present multicentre RCT was conducted in four centres in four European countries. The aim was to investigate whether the use of autofluorescence to detect the parathyroid glands during total thyroidectomy reduced the risk of postoperative hypoparathyroidism, defined by a low level of parathyroid hormone (PTH) on the day after surgery.

## Methods

The study was approved by the local ethical committees of participating centres (Swedish Ethical Review Authority: 2019-05948; Regional Committees for Medical and Health Research Ethics, REK sør-øst D: no. 93484; Bioethics Committee of Jagiellonian University: 1072.6120.286.2019; Ethical Committee City of Vienna: EK 21-172-0821). The study was registered with ClinicalTrials.gov (NCT04509011).

Patients referred to participating departments who could be included in the study received written and oral information. Informed written consent was obtained from all study patients.

### Inclusion and exclusion criteria

Included in the trial were patients older than 18 years with thyroid disease scheduled for total thyroidectomy, regardless of the preoperative diagnosis. Patients were excluded if: they had a history of thyroid or parathyroid surgery; were scheduled for concurrent parathyroid surgery; had renal insufficiency; were pregnant or breast feeding; were allergic to the contrast agent (iodine); were unable to understand the study information; or were unable to participate in the planned follow-up programme.

### Study outcomes

The primary outcome was the rate of low (below the normal range) plasma PTH level on the first day after surgery.

There were nine secondary outcomes: postoperative medication with vitamin D at discharge, defined as medication with active vitamin D—dihydrotachysterol (Anatomical Therapeutic Chemical code (ATC) A11CC02), alfacalcidol (ATC A11CC03), or calcitriol (ATC A11CC04)—owing to postoperative hypocalcaemia, registered in the Eurocrine^®^ database; postoperative medication with oral calcium at discharge, defined as medication with oral calcium—calcium carbonate (A12AA04) and calcium lactate gluconate (A12AA06)—registered in the Eurocrine^®^ database; intraoperative identification of parathyroid glands, recorded as number of glands identified during surgery; autotransplantation of parathyroid tissue; excision of parathyroid glands, recorded as number of excised parathyroid glands found in the specimen at histopathology; duration of surgery (skin to skin); duration of hospital stay; readmission owing to hypocalcaemia, defined as any subsequent hospital admission within 30 days for hypocalcaemia-related symptoms; and medical treatment for hypoparathyroidism lasting 6 months or longer.

### Eurocrine^®^ database

Eurocrine^®^, a pan-European database and quality register for endocrine surgery, was used to register the study data. The database is run by the Eurocrine Society, a not-for-profit organization based in Vienna (http://www.eurocrine.eu). The study was designed in My Eurocrine, a specific tool used for observational studies and RCTs within the Eurocrine^®^ database. Thus, except for core variables in the database, My Eurocrine was used to add specific study variables, screen for inclusion and exclusion criteria, and register patient consent. Participants were randomized automatically either to the intervention or control group after consent had been registered electronically in the database. Variables included in the present study are shown in the *supplementary material*.

### Autofluorescence

For detection of autofluorescence of the parathyroid glands in the intervention group, Fluobeam^®^ LX (Fluoptics, Grenoble, France) was used by all participating centres. Instructions and training in the use of Fluobeam^®^ LX was provided either face to face or virtually by representatives from Fluoptics. Experience in use of Fluobeam^®^ LX before commencing the study varied among the centres from none to several hundred procedures. The use of angiography with ICG for fluorescence during surgery was at the discretion of the surgeon. For patients in whom ICG angiography was used, the degree of ICG fluorescence was scored as suggested by Fortuny *et al.*^11,13^.

### Surgical technique

All patients had surgery with a standard Kocher incision and high extracapsular dissection of the thyroid gland. After division of the middle thyroid vein, the thyroid lobe was rotated medially. In the control group, parathyroid glands were identified and preserved meticulously whenever possible, but were not searched for, if not in the immediate operative field. After resection of the second lobe, the thyroid specimen was evaluated by the naked eye for any suspected parathyroid tissue.

In the intervention group, Fluobeam^®^ LX was used to identify spots with autofluorescence; if these were deemed to be of parathyroid origin, autofluorescence was used to guide the line of dissection to preserve the vascular supply. Fluobeam^®^ LX was used to evaluate the resected specimen during the operation. It was noted whether a parathyroid gland was identified first by the naked eye or by autofluorescence.

In both groups, autotransplantation of parathyroid tissue was performed at the discretion of the surgeon, and it was noted whether this was due to suspected ischaemia of the parathyroid gland or for anatomical reasons.

### Biochemistry

Analysis of total calcium and PTH levels was undertaken at the local laboratory. For the PTH analysis, three centres used the Elecsys^®^ PTH STAT, Instrument Cobas e602 (Roche Diagnostics, Rotkreuz, Switzerland). One participating centre used Abbot Alinity™ I Intact PTH CTL (Abbot Park, Illionois, US). Details of the instruments and assays that were used in the different centres, normal ranges, and precision of assays are provided in the *supplementary material*.

### Follow-up after surgery

Blood was drawn for analysis of PTH and total calcium levels the day after surgery. After operation, patients were treated for hypocalcaemic symptoms and biochemistry, and discharged according to local routine. Similarly, the first follow-up was carried out according to local routine. The date for follow-up was registered, as well as medical treatment for hypoparathyroidism with oral calcium and vitamin D. Levels of total calcium and PTH were measured. Patients with normal PTH and calcium levels, and who did not need treatment for hypoparathyroidism, were not followed further.

Patients treated for hypoparathyroidism were followed until medical treatment could be terminated or for at least 6 months. The date of last follow-up, and levels of PTH and calcium at last follow-up, were registered, as well as any medical treatment for hypoparathyroidism.

### Study considerations

This was a pragmatic study. The surgical procedure, including autotransplantation of parathyroid tissue as required, and treatment of biochemical or symptomatic hypocalcaemia, was undertaken according to local policies, reflecting a variety of contemporary clinical routines. The four centres that participated in the study are identified by letters A–D.

Postoperative PTH concentration is known to be the best predictor of postoperative hypoparathyroidism, with a sensitivity of 69–100 per cent in predicting hypocalcaemia^21^. A low PTH level (below the reference range) on the first postoperative day, was chosen as the primary outcome of the investigation. In previous observational studies^22–25^, postoperative low PTH levels ranged between 10 and 20 per cent.

Based on an α of 80 per cent and β of 5 per cent, it was calculated that 280 patients (140 in each arm) needed to be included to show a reduction from 15 to 5 per cent for the primary outcome, low level of PTH on the first postoperative day. With an expected recruitment of approximately 70 per cent of eligible patients, and allowing for drop-outs owing to change of surgical strategy before or during surgery, protocol violations, and loss to follow-up, at least 400 patients needed to be invited to participate.

Randomization was performed 1 : 1 in blocks of 10 through the Eurocrine^®^ platform to either operation with Fluobeam^®^ LX (intervention group) or surgery without (control group). The treatment allocation was concealed from the patient; the study was thus single-blinded.

### Study definitions

A low level of PTH was defined as a concentration below the reference range for the bioassay used as opposed to normal/high level. Clinically relevant biochemical hypocalcaemia was defined by a total calcium level below 2.00 mmol/l. Temporary hypoparathyroidism was defined by the need for treatment with a vitamin D analogue or oral calcium therapy after surgery to maintain a normal level of blood calcium, which was terminated within 6 months. Permanent hypoparathyroidism was defined by the need for treatment with a vitamin D analogue or oral calcium therapy for more than 6 months.

### Statistical analysis

Statistical analyses were undertaken in accordance with the protocol. Incorrectly randomized subjects with violation of one or more entry criteria were excluded from the analysis if the criterion violated had been measured and documented before randomization. Patients who had a unilateral procedure on clinical grounds (mostly owing to suspected intraoperative nerve injury), were excluded from the analysis, because these patients by definition had no risk of postoperative hypoparathyroidism.

The two-sided confirmatory null hypothesis for the primary endpoint and two-sided exploratory null hypotheses for other endpoints were analysed in between-group comparisons using Pearson’s χ^2^ test, or Fisher’s exact test if an expected frequency was lower than 5. The Cochran–Armitage trend test was used to test trends. Differences in continuous variables between groups were analysed using Student’s *t* test.

Estimated relative risks (RRs) are presented with 95 per cent confidence intervals, unstratified and stratified with Mantel–Haenszel weighting. Multivariable statistical models were computed using the proportional hazards model with constant follow-up and the Huber–White sandwich estimator to estimate RRs directly^26^. The statistical significance level was set at a type I error rate of 5 per cent. All multiple testing was exploratory and performed without multiplicity correction. All calculations were done in Stata^®^ release 17 (StataCorp, College Station, TX, USA).

## Results

**Fig. 1:**
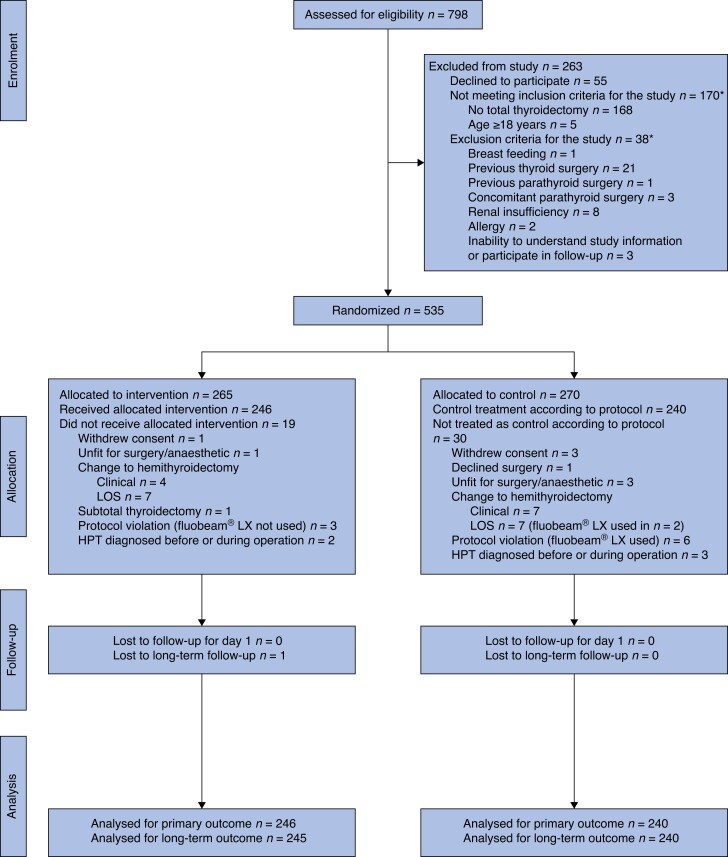
CONSORT diagram for trial *A patient could meet several exclusion criteria. LOS, loss of signal during intraoperative nerve monitoring. HPT, hyperparathyroidism.

The CONSORT diagram for the study is shown in *Fig. 1*. Patients were recruited between 10 January 2021 and 31 August 2022. Follow-up was complete on 28 February 2023. Some 798 patients were assessed for eligibility for the study, of whom 535 were randomized. Finally, 486 patients received the allocated intervention, 246 in the intervention group and 240 in the control group. The mean(s.d.) age of the patients was 50.8 (15.0) years and 389 (80.0 per cent) were women.

*Table 1* shows preoperative data for the two treatment groups, and *Table S1* shows preoperative data, results for the primary outcome, and number of surgeons involved in the study for the four participating centres. In short, the two groups were well balanced, but the number of included patients differed among the centres.

**Table 1:** Preoperative variables for 486 patients randomized to the use of autofluorescence to identify parathyroid glands (intervention) or to control group

	Intervention (*n* = 246)	Control (*n* = 240)
**Age (years), mean(s.d.)**	50.2(14.0)	51.4(15.9)
**Total calcium (mmol/l), mean(s.d.)**	2.38(0.08)	2.38(0.08)
**Sex ratio (F : M)**	200 : 46	189 : 51
**Indication for surgery**		
Compression	26 (10.6)	26 (10.8)
Exclusion of malignancy	110 (44.7)	89 (37.1)
Malignancy	55 (22.3)	61 (25.4)
Thyrotoxicosis	55 (22.3)	64 (26.7)
**Vitamin D supplements**	68 (27.6)	80 (33.3)
**Oral calcium supplement**	7 (2.8)	14 (5.8)
**Bisphosphonates**	1 (0.4)	2 (0.8)
**Lithium**	4 (1.6)	2 (0.8)

Values are *n* (%) unless otherwise indicated.

### Primary outcome

The numbers of patients with low levels of PTH on the first postoperative day were 64 (26.0 per cent) in the Fluobeam^®^ LX group and 77 (32.1 per cent) in the control group (*P* = 0.141; RR 0.81, 95 per cent c.i. 0.61 to 1.07) (*Table 2*).

**Table 2:** Outcome for 486 patients randomized to the use of autofluorescence (intervention) or to control group to identify parathyroid glands during thyroidectomy

	Intervention (*n* = 246)	Control (*n* = 240)	*P*†
**Low level of PTH on day 1**	64 (26.0)	77 (32.1)	0.141
**Serum calcium on day 1 (mmol/L), mean(s.d.)**	2.18 (0.14)	2.18 (0.13)	0.651‡
**Total calcium < 2.00 mmol/l on day 1**	26 (10.6)	22 (9.2)	0.604
**No. of parathyroid glands identified**			< 0.001
0	0	1	
1	5	11	
2	25	53	
3	70	98	
4	146	77	
**Intraoperative identification of parathyroid gland in specimen (*n* = 485)**	58 (23.6)	16 (6.7)	< 0.001
**Autotransplantation of parathyroid glands**	51 (20.7)	35 (14.6)	0.076
**Central lymph node clearance**	82 (33.3)	92 (38.3)	0.250
**Duration of operation (min)**			0.147
< 75	73 (29.7)	87 (36.2)	
75–120	80 (32.5)	81 (33.8)	
≥121	93 (37.8)	72 (20.0)	
**Hypocalcaemia treated with i.v. calcium in hospital**	4 (1.6)	3 (1.3)	1.00
**Oral calcium at discharge**	63 (25.6)	66 (27.5)	0.637
**Vitamin D at discharge**	48 (19.5)	54 (22.5)	0.419
**Duration of hospital stay (days), mean(s.d.) (*n* = 476)**	2.2(2.1)	2.1(0.9)	0.744‡
**Readmission within 30 days owing to hypocalcaemia**	5 (2.0)	3 (1.3)	0.724
**Parathyroid gland identified in specimen on histology (*n* = 485)**	18 (7.3)	50 (20.8)	< 0.001
**Treated for hypoparathyroidism at any time after surgery**	79 (32.1)	78 (32.5)	0.927
**Permanent (> 6 months) medically treated hypoparathyroidism**	6 (2.4)*	10 (4.2)	0.290

Values are *n* (%) unless otherwise indicated. *Based on 245 patients; 1 was lost to follow-up. PTH, parathyroid hormone; i.v., intravenous. †Pearson’s χ^2^ test or Fisher’s exact test, except ‡Student’s *t* test.

A subanalysis of the primary outcome showed that the rate of low PTH levels on the day after surgery was lower among patients who underwent central lymph node dissection in the Fluobeam^®^ LX group: 15 of 82 (18.3 per cent) *versus* 31 of 92 (33.7 per cent) (*P* = 0.021; RR 0.54, 0.31 to 0.93) (*Table 3*). Among patients aged 38 years or less, the rate of low PTH levels was also lower in the Fluobeam^®^ LX group than in controls (RR 0.48, 0.27 to 0.85) (*Table 3*). There was no difference in rate of low PTH in relation to indication for surgery, sex, or surgical centre among the two randomized groups of patients.

**Table 3:** Subanalysis of 141 patients with a low parathyroid hormone level on the day after thyroidectomy

	Proportion of patients with low PTH level among total cohort		P*
	Intervention	Control	
**Sex**			
M	7 of 46 (15.2)	13 of 51 (25.5)	0.212
F	57 of 200 (28.5)	64 of 189 (33.9)	0.254
**Age (years)**			
≤ 38	12 of 56 (21.4)	28 of 63 (44.4)	0.008
39–51	15 of 67 (22.4)	16 of 51 (31.4)	0.272
52–61	9 of 72 (26.4)	12 of 51 (23.5)	0.719
≥ 62	18 of 51 (35.3)	21 of 75 (28.0)	0.385
**Preoperative indication for surgery**			
Compression	4 of 26 (15.4)	8 of 26 (30.7)	0.188
Exclusion of malignancy	34 of 110 (30.1)	26 of 89 (29.2)	0.796
Malignancy	13 of 55 (23.6)	21 of 61 (34.4)	0.202
Thyrotoxicosis	35 of 119 (29.4)	22 of 64 (34.4)	0.200
**Central lymph node dissection**			
Yes	15 of 82 (18.3)	31 of 92 (33.7)	0.021
No	19 of 164 (29.9)	46 of 148 (31.1)	0.818
**Surgical centre**			
A	37 of 108 (34.3)	36 of 102 (35.3)	0.875
B	12 of 43 (27.9)	18 of 39 (46.2)	0.087
C	7 of 19 (36.8)	8 of 17 (47.1)	0.535
D	8 of 76 (10.5)	15 of 82 (18.3)	0.167

Values are *n* (%) unless otherwise indicated. PTH, parathyroid hormone. *Pearson’s χ^2^ test or Fisher’s exact test.

The rate of low PTH levels on the first postoperative day for the total group of patients differed among centres (centre A: 73 of 210 patients (34.8 per cent); centre B: 30 of 82 (36.6 per cent) patients; centre C: 15 of 36 (41.7 per cent); and centre D: 23 of 158 (14.6 per cent); *P* < 0.001). Furthermore, although all centres exhibited a decrease in rates of low PTH for the intervention group compared with the control group, this was not significant, and the impact varied among the centres (*Table S2*). As the number of included patients differed among centres (210, 82, 36, and 158 in centres A–D respectively), a multivariable sensitivity analysis was performed with inclusion of age, sex, indication for surgery, central lymph node dissection, duration of operation (as a proxy for trauma), study centre, and the use of autofluorescence during surgery. This analysis showed that there was an influence of study centre (D *versus* A: HR 0.24, 95 per cent c.i. 0.13 to 0.43), and sex (men: HR 0.62, 0.41 to 0.94), whereas the risk of a low level of PTH in the intervention group did not reach significance (HR 0.81, 0.62 to 1.06; *P* = 0.118) (*Table S3*).

The levels of total calcium on the day after surgery did not differ between the two patient groups, nor did the rate of calcium levels below 2.00 mmol/l (*Table 2*).

### Secondary outcomes

There was no difference between the intervention and control groups regarding duration of operation, central lymph node clearance, treatment with intravenous calcium, duration of hospital stay before discharge, and readmission within 30 days owing to hypocalcaemia (*Table 2*). Similarly, there was no difference between the groups in prescribed oral calcium and vitamin D medications at discharge. Interestingly, in patients who underwent central lymph node clearance, oral calcium was less often prescribed in the intervention group: 17 of 82 (20.7 per cent) *versus* 32 of 92 (34.8 per cent) (*P* = 0.040). Similarly, among patients who had central lymph node dissection, vitamin D was prescribed at discharge to 13 of 82 patients (15.9 per cent) in the intervention group compared with 28 of 92 (30.4 per cent) in the control group (*P* = 0.024). Oral calcium and vitamin D at discharge were prescribed less often at centre D (data not shown).

**Fig. 2:**
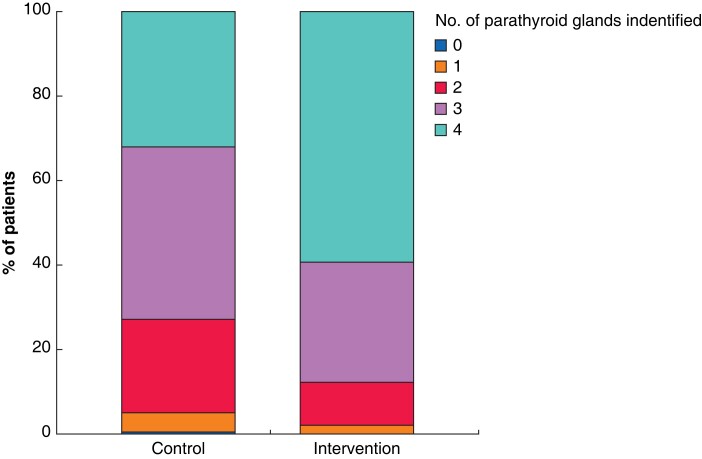
Number of parathyroid glands identified in control and intervention groups expressed as a percentage of patients

More parathyroid glands were identified during surgery in the Fluobeam^®^ LX group (*P* < 0.001) (*Fig. 2*), and, specifically, parathyroid glands were more likely to be detected in the surgical specimen during the procedure: 58 of 246 patients (23.6 per cent) in the Fluobeam^®^ LX group compared with 16 of 240 (6.7 per cent) in the control group (*P* < 0.001) (*Table 2*). Of the 58 parathyroid glands detected in the intervention group, the first detection was by autofluorescence for 50 glands and by the naked eye for 8.

Some 86 of 486 patients (17.7 per cent) had autotransplantation of at least 1 parathyroid gland, 51 of 246 (20.7 per cent) in the intervention group and 35 of 240 (14.6 per cent) in the control group (*P* = 0.076) (*Table 2*). Parathyroid tissue was autotransplanted into the muscle in all patients. Techniques used in the 86 patients were injection in 29 (33.7 per cent), and pieces in a single muscle pocket in 39 (45.3 per cent) or in several muscle pockets in 18 (20.9 per cent).

Conversely, on final histology, more parathyroid glands were detected in the specimen in the control group: 50 of 240 (20.8 per cent) *versus* 18 of 245 (7.3 per cent) (data missing for 1 patient) in the Fluobeam^®^ LX group (*P* < 0.001).

In the subanalysis of 174 patients who had central lymph node dissection, 6 of 92 (6.5 per cent) in the control group had a parathyroid gland identified in the surgical specimen during surgery compared with 28 of 82 (34.1 per cent) in the intervention group (*P* < 0.001). There was a pronounced difference in the number of parathyroid glands identified between the groups, with more than two glands being identified more often in the intervention group (*Fig. S1*). Importantly, in this subgroup, a parathyroid gland was found in the specimen on histology in 33 of 92 patients (35.9 per cent) in the control group compared with only 4 of 82 (4.9 per cent) in the intervention group (data missing for 1 patient) (*P* < 0.001).

Some 157 patients (32.3 per cent) were treated for hypoparathyroidism at some point after operation, 79 of 246 (32.1 per cent) in the intervention group (1 patient was lost to follow-up) and 78 of 240 (32.5 per cent) in the control group (*P* = 0.927).

Of the 157 patients treated for postoperative hypoparathyroidism, 104 (66.2 per cent) had a low level of PTH, and 53 had a normal or high PTH level on postoperative day 1. Conversely, of the 329 patients not treated for hypoparathyroidism, 37 (11.3 per cent) had a low PTH level, and 292 had a normal or high PTH level. The sensitivity and specificity for a low level of PTH to predict postoperative treatment of hypoparathyroidism at any time after surgery was 66.2 and 88.8 per cent respectively. The positive predictive value was 73.8 per cent.

One of the patients treated for hypoparathyroidism, who had been randomized to the intervention group, refused long-term follow-up. All other patients received regular follow-up examinations, with clinical and biochemical evaluation including analysis of calcium and PTH levels. Medical treatment could be terminated within 6 months in 140 of the 156 patients (89.7 per cent) who were followed up according to protocol, and these patients were classified as having temporary hypoparathyroidism. Medical treatment was terminated in 72 of 78 patients in the intervention group (92.3 per cent) and 68 of 78 (87.2 per cent) in the control group.

Some 16 patients were treated for hypoparathyroidism for at least 6 months, and thus defined as having permanent hypoparathyroidism according to the study protocol: 6 of 245 (2.4 per cent) in the intervention group and 10 of 240 (4.2 per cent) in the control group (*P* = 0.290). Data for these patients are summarized in *Table 4*. They all had a low level of PTH on postoperative day 1 and half had a calcium value below 2.00 mmol/l. The sensitivity and specificity for a low PTH value to predict permanent hypoparathyroidism was 100 and 73.6 per cent respectively. The positive predictive value was 11.4 per cent.

**Table 4:** Data for 16 patients treated for hypoparathyroidism for 6 months or more after total thyroidectomy

	No. of patients* (*n* = 16)
Age (years), mean(s.d.)	47.6(18.4)
Sex ratio (F : M)	11 : 5
No. in control group	10
Calcium < 2.00 mmol/l on day 1	8
Low PTH on day 1	16
Oral calcium at discharge	13
Vitamin D at discharge	13
Low PTH at last follow-up	8
Calcium < 2.15 mmol/l at last follow-up	10

Unless indicated otherwise. PTH, parathyroid hormone.

Angiography with ICG for fluorescence during surgery was used in only four patients. Two of these had a low level of PTH on the first day after surgery, and two were treated temporarily for postoperative hypoparathyroidism.

## Discussion

The main result of this RCT, which compared the use of Fluobeam^®^ LX autofluorescence to detect parathyroid glands during total thyroidectomy with a control group without monitoring of autofluorescence, was that the risk of low levels of PTH on the first day after operation did not differ between groups (26.0 *versus* 32.1 per cent respectively; *P* = 0.141; RR 0.81, 95 per cent c.i. 0.61 to 1.07). There was no difference in the rate of low levels of calcium (below 2.00 mmol/l) on the first postoperative day, nor in the rate of treatment with intravenous calcium during the hospital stay.

A subanalysis showed that the use of autofluorescence was, however, beneficial in patients who underwent central lymph node clearance; the rate of low levels of PTH on the first postoperative day was 8.3 per cent in the intervention group compared with 33.7 per cent in the control group (*P* = 0.021; RR 0.54, 0.31 to 0.93). Interestingly, in patients who had central lymph node clearance, oral calcium and vitamin D were less often prescribed at discharge in the intervention group than in the control group.

More parathyroid glands were detected during operation in the intervention group compared with the control group, including in the surgical specimen. Most glands in the specimen were detected by autofluorescence in the intervention group. In the intervention group, autotransplantation was performed in 51 of 286 patients (20.7 per cent) compared with 35 of 240 (14.6 per cent) in the control group (*P* = 0.076). Conversely, more parathyroid glands were detected in the control group on final histology. In agreement with the result of the subanalysis for the primary outcome, this result was even more pronounced in patients who underwent central lymph node dissection.

Because there was a difference in included patients, and in the rate of low PTH levels on the first postoperative day, between participating centres (*Table S1*), a sensitivity analysis was performed by including the use of autofluorescence, age, sex, indication for surgery, central lymph node dissection, duration of operation, and centre. This analysis confirmed the association between low PTH level and centre, whereas the HR for intervention group was 0.793 (95 per cent c.i. 0.607 to 1.036) (*Table S3*). The reason for the reduced risk of a low PTH level at centre D is not known. Although speculative, there may be an influence of surgical technique or surgeon volume, because only 2 surgeons performed all 158 operations at centre D (*Table S3*).

Some 157 patients were treated after surgery for hypoparathyroidism, 31.1 per cent in the intervention group and 32.5 per cent in the control group (*P* = 0.927). These patients were followed, including analysis of PTH and calcium levels, and the vast majority in both groups recovered. Sixteen patients, 6 (2.4 per cent) in the intervention group and 10 (4.2 per cent) in the control group (*P* = 0.290), were still being treated for hypoparathyroidism after 6 months, which in this study was defined as having permanent hypoparathyroidism. All these patients had a low level of PTH on day 1 after surgery.

These results are only partly in agreement with those of a multicentre study by Benmiloud *et al.*^15^. In the previous study, which had rate of calcium below 2.00 mmol/l on postoperative day 1 or 2 as its primary outcome, the rate of low calcium differed between the two groups (9.1 per cent in the intervention group *versus* 21.7 per cent in the control group). In the present study, the rate of calcium below 2.00 mmol/l was 10.6 per cent in the intervention group *versus* 9.2 per cent in the control group on the first postoperative day. Similarly, three other single-centres RCTs^16,17,19^ did not find a difference in postoperative calcium values on postoperative day 1. The discrepancy between the present results and those of Benmiloud *et al.*^15^, is not clear, because the inclusion criteria for patients in both studies were quite similar. A possible explanation is that, for uniformity, sampling for calcium and PTH was undertaken on the day after surgery in the present study because many patients are discharged on the first postoperative day. Another explanation is that, in the study by Benmiloud *et al.*^15^, 81 per cent of the patients were operated at one of the three French participating centres, whereas the present pragmatic study involved multiple centres and surgeons in several countries.

Postoperative calcium levels depend on several factors, including preoperative calcium level, the level of 25-hydroxyvitamin D, thyrotoxicosis^21^, and longer duration of operation^27^. Additionally, any self-medication with vitamin D and calcium supplements may act as confounders.

A low PTH level on the first postoperative day was chosen as primary outcome in the present study because postoperative PTH concentration is considered the earliest and most sensitive predictor of postoperative hypoparathyroidism^28,29^. Postoperative measurement seems to be more sensitive than intraoperative measurement. Different time points for PTH measurement have been used within the first 24 h after surgery, with no significant difference between them, and with a sensitivity of almost 90 per cent and a specificity above 95 per cent^30^. Furthermore, the lower limit of the PTH assay leads to a high sensitivity and specificity for predicting hypoparathyroidism^30^. In the present study, the sensitivity of a low level of PTH in predicting medical treatment for hypoparathyroidism was moderate (just below 70 per cent), albeit with a high sensitivity (almost 90 per cent). Conversely, for treatment of hypoparathyroidism after 6 months, the sensitivity was 100 per cent, but with a modest specificity of approximately 75 per cent. The reason for the lower sensitivity in predicting postoperative medical treatment of hypoparathyroidism is not known, but the main factor could be that this was a pragmatic multicentre study in which treatment for hypoparathyroidism was according to local routine.

It may be argued that the measured PTH level should be evaluated in the context of the measured calcium level; that is, a patient could present with hypoparathyroidism with a normal PTH level. There is, however, no firm accepted definition of an inappropriately low PTH level in the face of hypocalcaemia.

In the present study, the difference in the rate of low PTH on the first postoperative day between the intervention and control groups failed to reach statistical significance. Broadly in agreement, there was also no difference in PTH levels on the day after surgery between the intervention and control groups in other RCTs^15–17,19^. A subanalysis of patients undergoing central lymph node dissection, however, showed an almost halved rate of low levels of PTH on postoperative day 1 in the intervention group. This seems logical given the well known problem in identifying and protecting the parathyroid glands during clearance in the central compartment, not only of malignant nodes but also as part of a prophylactic lymph node dissection^31^.

The power calculation for the primary outcome in this study was based on the results from observational studies^22–25^. The present results showed that the rate of low levels of PTH on postoperative day 1 was clearly underestimated (26.0 and 32.1 per cent in the intervention and control groups respectively). To show a difference at the 5 per cent level with 80 per cent power, approximately 1800 patients would need to be randomized. This would be a big task, although not impossible. Furthermore, the rate differed between 14.6 and 41.7 per cent among the centres. A sensitivity analysis including age, sex, use of autofluorescence, indication for surgery, central lymph node dissection, duration of operation, and study site confirmed the influence of the individual centre. Thus, to evaluate the impact of autofluorescence in detecting parathyroid glands on the risk of hypoparathyroidism, multicentre studies are clearly needed, preferably RCTs; alternatively, prospective observational studies involving large numbers of patients, from a variety of departments, are required.

In agreement with previous studies^15,19^, more parathyroid glands were identified during surgery in the intervention group than in the control group. Inadvertent excision of parathyroid glands was reported more often by the pathologist on final histology in the control group, also in agreement with Benmiloudet *et al.*^15^ and Rossi *et al.*^19^. Bellier *et al.*^32^ showed that the frequency of inadvertent excision of parathyroid glands with the help of autofluorescence was reduced to 6.5 per cent, almost the same as in the present study. This should logically lead to a higher rate of autotransplantation in the autofluorescence group. Although the rate of autotransplantation of parathyroid tissue was 20.7 per cent in the intervention group compared with 14.6 per cent in the control group, the difference did not reach statistical significance (*P* = 0.076). The reason for the discrepancy between parathyroid glands identified during surgery and number of glands transplanted in the intervention group is not known. One explanation could be the intraoperative uncertainty of the histology of glands identified by autofluorescence, which could be especially of concern during operations for thyroid cancer. However, one study^32^ suggested that, by using magnifying glasses, the surgeon may be able to differentiate autofluorescence of parathyroid origin from autofluorescence exhibited by other tissues.

There was no difference in the number of patients treated for hypoparathyroidism after surgery between the two groups (32.1 per cent in intervention group and 32.5 per cent in control group). The decision to treat hypoparathyroidism was based on local hospital guidelines, which indeed may have differed considerably. The patients were, however, followed clinically and with measurement of calcium and PTH levels until these normalized, although one patient refused follow-up. In most patients, medical treatment of hypoparathyroidism could be terminated. Some 16 patients were still being treated for hypoparathyroidism after 6 months, 6 (2.4 per cent) in the intervention group and 10 (4.2 per cent) in the control group. The 6-month threshold is well established; however, it is known that some patients may recover beyond that time point^33^, although, importantly, the secretory reserve of the parathyroid glands in such patients is not known.

This RCT was performed at the time of the COVID-19 pandemic, which proved be very challenging. The epidemiological situation with restrictions for patients and hospitals, including lockdowns, travel restrictions, and restrictions on the types of operation performed, varied greatly over time and among countries and centres. Delay in delivery of the Fluobeam^®^ LX equipment meant that the study started at different times in the participating centres. Hence, the number of patients varied considerably between centres, although there was no indication that this influenced the combined results (*Table S1*). Furthermore, local restrictions over time meant that it was extremely difficult to predict how many patients needed to be randomized to be able to accrue a certain number of operations. There was a considerable risk of an increased drop-out rate owing to the waiting time for surgery. Finally, as the pandemic subsided, more patients than anticipated were included in the study.

The number of surgeons involved varied between the centres, but this probably did not influence the combined results. Furthermore, experience with the Fluobeam^®^ LX device had no clear correlation with outcome.

In the context of these limitations, it can be concluded that the use of autofluorescence of the parathyroid gland, here employing Fluobeam^®^ LX, did not decrease the risk of a low level of PTH for the whole patient group, but decreased the risk among patients who underwent central lymph node dissection. Autofluorescence enabled more parathyroid glands to be identified during surgery and fewer parathyroid glands to be excised inadvertently, which is a very important benefit of the technique. The potential added value of angiography for ICG fluorescence needs to be investigated further as this was used in only four patients in the present study but has been suggested to be a valuable adjunct^14^.

## Supplementary Material

znad278_Supplementary_Data

## Data Availability

Data are available for non-commercial researchers by application to the corresponding author.
