# Supplementary material for: Impact of autofluorescence for detection of parathyroid glands during thyroidectomy on postoperative parathyroid hormone levels: parallel multicentre randomized clinical trial
Source: Br J Surg. 2023 Sep 27;110(12):1824–33. doi: 10.1093/bjs/znad278 (PMC10638529; doi:10.1093/bjs/znad278)

**Impact of auto-fluorescence for detection of parathyroid glands during thyroidectomy on postoperative PTH levels; a parallel randomized controlled multi-centre trial**

Bergenfelz A^1^, Barczynski M^2^, Heie A^3^ , Muth A^4,5^, Passler C^6^, Schneider M^6^, Wierzbicka P^2^, Konturek A^2^ Brauckhoff K^3,7^, Elf A-K^4,5^, Dahlberg J ^4,5^, Hermann M^6^

1. Department of Clinical Sciences-Lund, Medical Faculty, Lund University, Lund, Sweden
2. Department of Endocrine Surgery, Third Chair of General Surgery, Jagiellonian University Medical College, Krakow, Poland
3. Department of Breast- and Endocrine surgery, Haukeland University Hospital, Bergen, Norway
4. Department of Surgery, Sahlgrenska University Hospital, Gothenburg, Sweden
5. Department of Surgery, Institute of Clinical Sciences, Sahlgrenska Academy, University of Gothenburg, Gothenburg, Sweden
6. Department of Surgery, Klinik Landstraße, Wiener Gesundheitsverbund, Vienna, Austria
7. Department of Clinical Science, University of Bergen, Bergen, Norway

**Corresponding author.**

Correspondence:

Anders Bergenfelz

Professor emeritus

Department of Clinical Sciences-Lund

Medical Faculty

Lund University

[anders.bergenfelz@med.lu.se](mailto:anders.bergenfelz@med.lu.se)

+46 70 377 2086

**ORCID:**

**0000-0002-8355-6025**

**Supplementary Materials - Index**

| **Supplementary Methods** |  |
| --- | --- |
| Suppl. Methods 1 | *pag.7* |
| Suppl. Methods 2 | *pag. 11* |
| **Supplementary Figures and Tables** |  |
| Suppl. Table 1 | *pag. 14* |
| Suppl. Table 2  Suppl. Table 3  Suppl. Fig 1 | *pag. 15*  *pag 15*  *pag.17* |
|  |  |

**Supplementary Methods**

**Suppl.Methods 1**

*Variables analysed in the current randomized controlled trial.*

Preoperative:

Study centre

Group allocation

Age

Sex

Indication for surgery

Preoperative calcium value

Preoperative laryngoscopy

Preoperative treatment with oral calcium supplement

Preoperative treatment with Vitamin D supplement

Preoperative treatment with Bisphosphonates,

Preoperative treatment with Lithium

Preoperative PTH value

Preoperative Calcium value

Intra operative:

Type of thyroid operation

Operation time

Lymph node dissection (and type)

Identified parathyroid glands, number

With auto fluorescens

With the naked eye

Identified parathyroid glands on the specimen

Auto-transplanted parathyroid glands

Reason for transplantation

Site

Technique

ICG used or not used

Postoperative:

IV calcium during hospital stay

PTH day 1

Calcium day 1

Hospital stay (days)

Vitamin D analogue therapy at discharge (within 7 days)

Oral calcium at discharge (within 7 days)

First follow-up:

Date for follow-up

Hypocalcaemia related admissions to hospital within 30 days

Histological diagnosis

Weight of specimen

Parathyroid gland on the specimen

Calcium value

PTH value

Oral calcium treatment

Vitamin D analogue therapy

Long term follow-up:

Date for follow-up

Calcium value

PTH value

Oral calcium treatment

Vitamin D analogue therapy

**Suppl. Methods 2**

*Biochemical assays and normal values for the four centres participating in the current study.*

**Study site A**

PTH assay

Elecsys PTH Stat cobas e411 platform. Roche Diagnostics

Limit of detection: 0.127 pmol/L

Normal range: 1.60 – 6.90 pmol/L

Intra assay variation 1.8 %

Inter assay variation 2.85 %

Calcium assay

Cobas c702 (Roche Diagnostics)

Normal ranges:

> 20 years: 2,15-2,50 mmol/L

> 60 years: 2,20- 2,55 mmol/l

> 90 years: 2,05 – 2,40 mmol/l

**Study site B**

PTH Assay

Elecsys PTH STAT. Instrument Cobas e602. Roche Diagnostics

Limit of detection: 0.127 pmol/L

Normal range: 1.6-6.9 pmol/L

Intra assay variation: level 2.2, 2%; level 11.8, 1.4 %

Inter assay variation: level 2, 3.3%; level 8.5, 2.2%

Calcium assay

Cobas c702. Roche Diagnostics

> 20 years: 2.20-2.55 mmol/L

**Study site C**

PTH assay

Abbot Alinity i

Limit for detection: 0.11 ng/L

Normal range: 1.8-11 pmol/L

Intra assay variation: level 3, 7%; level 10, 3%

Inter assay variation: level 3.4, 4%; level 15.2, 3 %

Calcium assay

Alinity C

Normal range 2.15-2.50 mmol/L

**Study site D**

PTH Assay

Elecsys PTH (1-84). Cobas e 601. Roche Diagnostics

Limit of detection: 5.5 pg/ml

Normal range: 10 – 65 pg/ml

Calcium Assay

Cobas c 501 (Roche Diagnostics)

Normal range: 18­-60 years: 2.15-­2.50 mmol/L; 60­-90 years: 2.20­-2.55 mmol/L

**Supplementary Figures and Tables**

**Suppl. Table 1**

Preoperative variables, number of involved surgeons and primary outcome for patients randomized to the use of Fluobeam LX and to control for the four participating centres.

| Variable | Study centre A  N=210 | Study centre B  N=82 | Study centre C  N=36 | Study centre D  N=158 | P-value |
| --- | --- | --- | --- | --- | --- |
| Control/intervention total, number | 102/108 | 39/43 | 17/19 | 82/76 | 0.889 |
| Control/intervention for women, number | 80/86 | 27/33 | 16/16 | 66/65 | 0.916 |
| Control/intervention for age groups, number  *< 39 years*  *40-65 years*  *>66 years* | 22/21  63/68  17/19 | 15/14  17/27  7/2 | 9/8  5/8  3/3 | 21/16  36/50  25/10 | 0.963  0.632  0.122 |
| Indication for surgery  Control/intervention, number  *Compression symptoms*  *Excluding malignancy*  *Malignancy*  *Thyreotoxicosis* | 3/5  57/72  2/2  40/29 | 3/1  2/2  25/28  9/21 | 1/3  0/1  3/1  13/14 | 19/17  30/35  31/24  2/0 | 0.456  0.820  0.625  0.313 |
| Number of Surgeons in | 14 | 4 | 9 | 2 | NA |
| Low level* of PTH postoperative day 1 | 73/210 | 30/82 | 15/36 | 23/158 | <0.001 |

*As opposed to normal/high

NA= not applicable

**Suppl. Table 2.**

Analysis of the primary outcome variable, the rate of low PTH on the first postoperative day, for the four study centres. Numbers and percentages for the two groups of patients are shown.

**Variable Intervention Control p-value**

Low PTH/Total (%) Low/Total (%)

Centre A (n=210) 37/108 (34.3) 36/102 (35.3) 0.875

Centre B (n=82) 12/43 (27.9) 18/39 (46.2) 0.087

Centre C (n=36) 7/19 (36.8) 8/17 (47.1) 0.535

Centre D (n=158) 8/76 (10.5) 15/82 (18.3) 0.167

**Suppl. Table 3**

Sensitivity analysis of the risk for a low level of PTH on postoperative day one for patients randomized to the use of auto-fluorescens to detect parathyroid glands (intervention) or to control. Hazard ratio and 95 per cent confidence interval is shown.

| Variable | Hazard ratio | Lower confidence interval | Upper confidence interval | P-value |
| --- | --- | --- | --- | --- |
| **Ref. Control**  Intervention | 0.806 | 0.615 | 1.056 | 0.118 |
|  |  |  |  |  |
|  |  |  |  |  |
| **Ref. <38 years**  39-51 years  52-61 years  >62 years | 0.845  0.774  0.943 | 0.574  0.520  0.648 | 1.243  1.148  1.152 | 0.392  0.207  0.784 |
| **Ref. Women**  Men | 0.620 | 0.409 | 0.939 | 0.024 |
| **Ref. Compression**  Excluding malignancy  Malignancy  Thyreotoxicosis | 0.843  0.809  0.611 | 0.482  0.404  0.338 | 1.476  1.619  1.104 | 0.551  0.549  0.103 |
| **Ref. No lymph node dissection**  Central lymph node dissection | 1.395 | 0.921 | 2.112 | 0.116 |
|  |  |  |  |  |
| **Ref. Operation time <75 min**  75-120 min  >120 min | 0.773  0.729 | 0.476  0.428 | 1.255  1.243 | 0.297  0.246 |
| **Ref. study site A**  Site B  Site C  Site D | 0.993  1.300  0.235 | 0.604  0.809  0.129 | 1.634  2.089  0.429 | 0.979  0.279  <0.001 |

Ref.= referent

**Suppl. Fig.1**

Number of identified parathyroid glands intra-operatively in the sub-group of 174 patients undergoing central lymph node dissection. Per cent of total numbers is shown.

More than two parathyroid glands were identified in 93.9 per cent in the intervention group compared with 76.0 per cent in the control group.


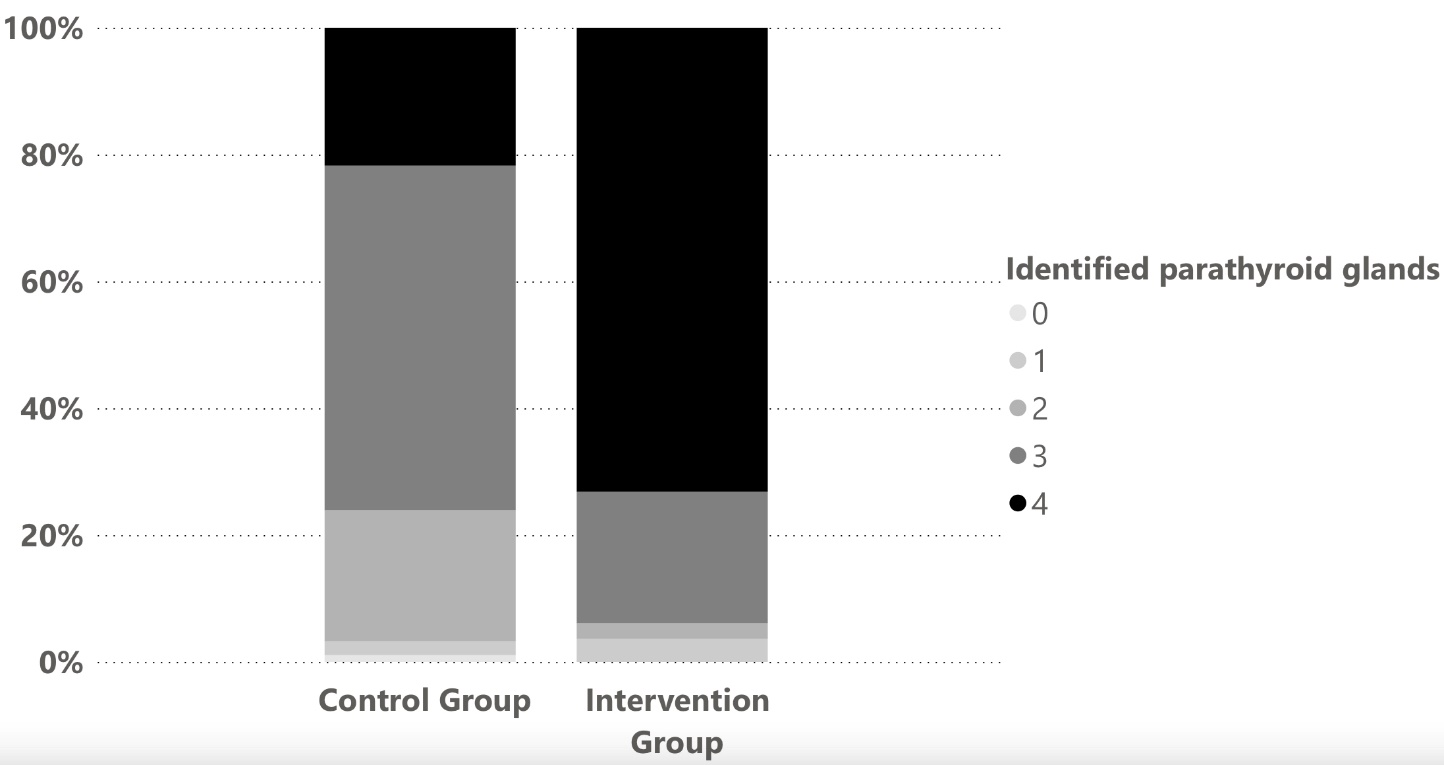

Supplement: znad278_Supplementary_Data [file znad278_supplementary_data.docx]
